# C2CDB: an advanced platform integrating comprehensive information and analysis tools of cancer-related circRNAs

**DOI:** 10.1093/bioadv/vbae112

**Published:** 2024-08-16

**Authors:** Yuanli Zuo, Wenrong Liu, Yang Jin, Yitong Pan, Ting Fan, Xin Fu, Jiawei Guo, Shuangyan Tan, Juan He, Yang Yang, Zhang Li, Chenyu Yang, Yong Peng

**Affiliations:** Laboratory of Molecular Oncology, Frontiers Science Center for Disease-related Molecular Network, State Key Laboratory of Biotherapy and Cancer Center, West China Hospital, Sichuan University, Chengdu 610064, China; Laboratory of Molecular Oncology, Frontiers Science Center for Disease-related Molecular Network, State Key Laboratory of Biotherapy and Cancer Center, West China Hospital, Sichuan University, Chengdu 610064, China; Laboratory of Molecular Oncology, Frontiers Science Center for Disease-related Molecular Network, State Key Laboratory of Biotherapy and Cancer Center, West China Hospital, Sichuan University, Chengdu 610064, China; Laboratory of Molecular Oncology, Frontiers Science Center for Disease-related Molecular Network, State Key Laboratory of Biotherapy and Cancer Center, West China Hospital, Sichuan University, Chengdu 610064, China; Laboratory of Molecular Oncology, Frontiers Science Center for Disease-related Molecular Network, State Key Laboratory of Biotherapy and Cancer Center, West China Hospital, Sichuan University, Chengdu 610064, China; Laboratory of Molecular Oncology, Frontiers Science Center for Disease-related Molecular Network, State Key Laboratory of Biotherapy and Cancer Center, West China Hospital, Sichuan University, Chengdu 610064, China; Laboratory of Molecular Oncology, Frontiers Science Center for Disease-related Molecular Network, State Key Laboratory of Biotherapy and Cancer Center, West China Hospital, Sichuan University, Chengdu 610064, China; Laboratory of Molecular Oncology, Frontiers Science Center for Disease-related Molecular Network, State Key Laboratory of Biotherapy and Cancer Center, West China Hospital, Sichuan University, Chengdu 610064, China; Laboratory of Molecular Oncology, Frontiers Science Center for Disease-related Molecular Network, State Key Laboratory of Biotherapy and Cancer Center, West China Hospital, Sichuan University, Chengdu 610064, China; Laboratory of Molecular Oncology, Frontiers Science Center for Disease-related Molecular Network, State Key Laboratory of Biotherapy and Cancer Center, West China Hospital, Sichuan University, Chengdu 610064, China; Laboratory of Molecular Oncology, Frontiers Science Center for Disease-related Molecular Network, State Key Laboratory of Biotherapy and Cancer Center, West China Hospital, Sichuan University, Chengdu 610064, China; Laboratory of Molecular Oncology, Frontiers Science Center for Disease-related Molecular Network, State Key Laboratory of Biotherapy and Cancer Center, West China Hospital, Sichuan University, Chengdu 610064, China; Laboratory of Molecular Oncology, Frontiers Science Center for Disease-related Molecular Network, State Key Laboratory of Biotherapy and Cancer Center, West China Hospital, Sichuan University, Chengdu 610064, China

## Abstract

**Motivation:**

Circular RNAs (circRNAs) play important roles in gene expression and their involvement in tumorigenesis is emerging. circRNA-related database is a powerful tool for researchers to investigate circRNAs. However, existing databases lack advanced platform integrating comprehensive information and analysis tools of cancer-related circRNAs.

**Results:**

We developed a comprehensive platform called CircRNA to Cancer Database (C2CDB), encompassing 318 158 cancer-related circRNAs expressed in tumors and adjacent tissues across 30 types of cancers. C2CDB provides basic details such as sequence and expression levels of circRNAs, as well as crucial insights into biological mechanisms, including miRNA binding, RNA-binding protein interaction, coding potential, base modification, mutation, and secondary structure. Moreover, C2CDB collects an extensive compilation of published literature on cancer circRNAs, extracting and presenting pivotal content encompassing biological functions, underlying mechanisms, and molecular tools in these studies. Additionally, C2CDB offers integrated tools to analyse three potential mechanisms: circRNA-miRNA ceRNA interaction, circRNA encoding, and circRNA biogenesis, facilitating investigators with convenient access to highly reliable information. To enhance clarity and organization, C2CDB has meticulously curated and integrated the previously chaotic nomenclature of circRNAs, addressing the prevailing confusion and ambiguity surrounding their designations.

**Availability and implementation:**

C2CDB is freely available at http://pengyonglab.com/c2cdb.

## 1 Introduction

Circular RNAs (circRNAs) are a class of single-stranded covalently closed endogenous RNA molecules that are generated by back-splicing, a process that the 5′ splice donor is connected to the upstream 3′ splice acceptor (Li *et al.* 2020). Most circRNAs are composed of exonic sequences (exonic circRNAs, EcRNAs), while a small portion of circRNAs originates from intronic regions (circular intronic RNAs, ciRNAs) or both exonic and intronic regions (exon intron circRNAs, EIciRNAs).

Emerging evidence has demonstrated that circRNAs exert vital biological functions through sponging miRNA, serving as decoys for RNA-binding protein (RBP) or translating into peptides (Li *et al.* 2020). For example, a circRNA named CDR1as harbors 63 conserved binding sites of miR-7, and it functions to bind miR-7 with Argonaute proteins, thus suppressing miR-7 activity (Memczak *et al.* 2013). circITGB6 was reported to enhance the mRNA stability of PDPN gene by directly interacting with IGF2BP3 (Li *et al.* 2023). Recently, circRNAs are found to be dysregulated in cancers. We profiled circRNA expression patterns in several cancer types and revealed that cancers not only share common expression patterns of circRNAs, but also exhibit distinct circRNA expression signatures (Wang *et al.* 2019, 2022). Moreover, dysregulated circRNAs play crucial regulatory roles in the development and progression of tumors. For example, circNEIL3, a TGF*β*-repressive circRNA, inhibited tumor metastasis through recruiting the E3 ubiquitin ligase Nedd4L to degrade YBX1 (Chen *et al.* 2023). The peptide C-E-Cad encoded by the circRNA called circ-E-Cad promoted glioblastoma progression *via* stimulating EGFR-STAT3 signaling (Gao *et al.* 2021). However, biological functions and the underlying mechanisms of most circRNAs remain elusive.

To date, several circRNA databases have been developed to provide information about circRNA expression profiling and putative mechanisms. circBase is one of the earliest and widely-used circRNA database (Glažar *et al.* 2014), but it only provides basic circRNA information, such as sequences and expression patterns, and has not been updated for a long time. Although several databases, such as circMine (Zhang *et al.* 2022), circExp (Zhao *et al.* 2021), CIRCpedia2 (Dong *et al.* 2018), and MiOncoCirc (Vo *et al.* 2019), provide circRNA expression profiles in different types of tumors, they lack paired adjacent normal tissues, which hinders the identification of significantly differential circRNAs. CSCD2 (Feng *et al.* 2022), circBank (Liu *et al.* 2019), circAtlas (Wu *et al.* 2020), CircRic (Ruan *et al.* 2019), and circNet (Chen *et al.* 2022) databases provided the tools to analyze the potential mechanisms of circRNAs including the interactions of circRNA-miRNA and circRNA-RBP, but without detailed information and visualization results. Therefore, current databases and platforms cannot meet needs for cancer-related circRNA researchers. Most recently, lots of high-throughput sequencing data are generated, which could be utilized to identify more novel circRNAs, so it is important to incorporate such information in database.

Here, we developed a comprehensive platform called CircRNA to Cancer Database (C2CDB), providing expression profiles of circRNAs from paired normal and tumor tissues across up to 30 types of cancers. C2CDB has quantified a total of 3 18 158 cancer-related circRNAs. Notably, 96 597 of these circRNAs have not been reported in other databases. We also included the m^6^A modification site and secondary structure of circRNA, as well as the visualization tools in C2CDB. Furthermore, we collected detailed information of experimentally validated cancer-related circRNAs in published papers, such as circRNA functions, mechanisms, primers, siRNAs/shRNAs, and probes. Overall, C2CDB can serve as a useful resource for researchers to explore the expression profile, function, and mechanism of circRNA in cancers.

## 2 Methods

### 2.1 Data collection and processing

We collected transcriptome and microarray expression data from NCBI GEO by searching keywords “cancer” or “carcinoma”. Then we manually reviewed all available datasets and screened datasets using following criteria: (i) the library of transcriptome data was constructed using non-polyadenylated enrichment strategy; (ii) the samples with both tumor and paired normal adjacent tissues; (iii) all individuals did not receive any therapy prior to enrollment. Finally, 81 transcriptome and 53 microarray datasets were selected and downloaded. Another 10 transcriptome datasets generated by our previous study (Wang *et al.* 2022) were also enrolled (Supplementary Table S1). Besides, we also downloaded transcriptome data of non-tumor tissues and cells, and cancer cell-lines from NCBI GEO and ENCODE (Supplementary Tables S2–S4).

All datasets were processed using a unified pipeline. Firstly, FastQC (v0.11.8) was used to evaluate data quality, and Trimmomatic (v0.36) (Bolger *et al.* 2014) was applied to trim adapter sequences and remove low-quality reads.

### 2.2 circRNA identification from RNA-seq

We applied STAR (v2.5.3a) (Dobin *et al.* 2013) to align clean reads against the GRCh37.p13 reference genome. Then, CIRCexplorer2 (v2.3.8) (Zhang *et al.* 2016) was employed to identify circRNAs. The expression of circRNAs was normalized as the number of back-spliced RPM (reads per million mapped reads). After that, we kept circRNAs with an RPM greater than 1 in at least 10% of the samples for further analysis.

### 2.3 circRNA microarray dataset processing

For circRNA microarray datasets, 48 datasets were retrieved and processed using the R package GEOquery (v2.58.0) following the package’s instruction, while the remaining five datasets that failed to be processed by GEOquery were directly used the processed expression profile that uploaded by the authors (Davis and Meltzer 2007). The normalized expression profile of circRNA probes were then extracted from the results. To map the circRNA probe to a known circRNA, we downloaded the complete annotation information of circRNA microarray platforms from NCBI GEO, and then extracted the probe sequence. The 80 bp fragments on both sides of the circRNA back-splicing junction were extracted from the circBase database (Glažar *et al.* 2014). We then aligned the probe to the prepared circRNA back-splicing junction sequence to obtain the circBase ID corresponding to the probe.

### 2.4 Differential expression analysis

Wilcoxon rank-sum test was applied to identify the differentially expressed genes between tumor and normal samples, and the fold-change is calculated by dividing the average RPM expression of circRNA in tumor samples by its average RPM expression in normal samples. CircRNAs with average fold-change greater than 2 or less than 0.5, and *P* value less than .05 were considered as significantly differentially expressed.

### 2.5 Transcriptome analysis

The clean reads were mapped to the GRCh37.p13 reference genome using HISAT2 (v2.1.0) (Kim *et al.* 2019). StringTie (v2.0.4) (Pertea *et al.* 2015) was utilized to estimate the abundance of genes.

### 2.6 circRNA annotation

We extracted the genomic location, sequence, exon composition, cognate linear transcript, and host gene of circRNA based on the annotation results of CIRCexplorer2. Besides, we have manually curated circRNA literatures to collect the experimental data of circRNA, including biological function, mechanism, and molecular tools like RT-qPCR primer set, Northern blot probe, and siRNA/shRNA sequence. To obtain the IDs and aliases of circRNA in other circRNA databases, the list and annotation information of circRNAs were collected from the circBase (Glažar *et al.* 2014), circBank (Liu *et al.* 2019), circAtlas (Wu *et al.* 2020), DeepBase3 (Xie *et al.* 2021), CIRCpedia2 (Dong *et al.* 2018), exoRBase2 (Lai *et al.* 2022), and circRNADb (Chen *et al.* 2016) databases. The genomic position of circRNAs in the circAtlas and exoRBase2 databases was converted to the hg19 genome build using the UCSC liftOver tool (Nassar *et al.* 2023). We then overlapped the identified circRNAs with circRNA databases based on circRNA genomic position.

### 2.7 Epigenetic modification sites on circRNA

We collected five types of RNA modifications (m^6^A, m^5^C, m^1^A, m^7^G, and Psi) from m^6^A-Atlas database (Ruan *et al.* 2019). We utilized the bedtools intersect tool to map epigenetic modification sites to circRNA.

### 2.8 Somatic mutation sites on circRNA

The information of somatic mutation site (single nucleotide variant, SNV) was retrieved from COSMIC (Tate *et al.* 2019) database. The bedtools intersect tool was employed to map mutation sites to circRNA.

### 2.9 Prediction of circRNA secondary structure

The minimum free energy (MFE) secondary structure of circRNA was predicted using RNAfold (v2.4.14) (Lorenz *et al.* 2011). The base-pairing probability and spatial relative position of bases were extracted from the outputs of RNAfold. The schematic diagram of circRNA MFE structure was visualized using R package ggplot2.

### 2.10 Prediction of miRNA binding sites on circRNA

The mature sequences of miRNAs were downloaded from miRBase database (Kozomara *et al.* 2019). The miRNA binding sites on circRNAs were predicted by miRanda (v3.3a) with parameters -go -8 -ge -2 -sc 120 -strict (Enright *et al.* 2003). The sequence alignment between circRNA and miRNA was extracted using an in-house script.

### 2.11 Analysis of RBP binding sites on circRNA

To obtain the RBP binding sites on circRNAs, we have collected 1053 CLIP-Seq datasets of 291 RBPs from GEO, ENCODE, and starBase databases (Supplementary Table S5). The RBP binding sites on circRNAs were determined using the RBP binding sites that identified by CLIP-Seq datasets. Briefly, these identified RBP binding sites were firstly clustered and merged based on genomic coordinate to obtain the overlapped part of stacked binding sites among datasets. The resultant stacked binding sites were mapped to circRNAs to obtain the relative position of RBPs on circRNA using bedtools intersect tool. The binding motifs of 178 RBPs were collected from beRBP (Yu *et al.* 2019) and RBPmap (Paz *et al.* 2014) databases. Then FIMO (Grant *et al.* 2011) was utilized to predict RBP binding sites on circRNAs based on collected RBP motifs with the –thresh 1e−4 parameter.

### 2.12 Assessment of circRNA coding potential

We applied an in-house script to predict all putative open reading frames (ORFs) within circRNAs and kept the ORFs with a minimum length of 20 amino acids. Considering that circRNA translation is driven by IRES (internal ribosome entry site) element or m^6^A modification, we firstly retrieved experimentally verified IRES sequences from the IRESbase database (Zhao *et al.* 2020) and m^6^A modification sites detected by m^6^A-seq and related techniques from the m^6^A-Atlas (Tang *et al.* 2021). Then, circRNA sequences were aligned to IRES sequences to identify putative IRES element within circRNA using blastn (v2.11.0) with at least 90% sequence identity and a cutoff 30 nucleotides alignment length. Besides, the relative position of m^6^A modification sites on circRNA were determined by mapping circRNA to the collected m^6^A sites using bedtools intersect tool. We kept the circRNAs with putative IRES element or m^6^A modification sites as possible translatable circRNAs. We then annotated these potential translatable circRNAs using publicly available translatome datasets to provide direct *in vivo* translation evidence for these circRNAs (Supplementary Table S6).

### 2.13 Web interface implementation

The web interfaces were built on an Apache2 Server. The front-end design was created using HTML5 and CSS3 to arrange the form layout. For data transfer and interactive functions, the JQuery JavaScript library was utilized. Additionally, various extension plug-ins were incorporated to enhance the interactive development. Data visualization was achieved through the utilization of ECharts (https://echarts.apache.org) and HighCharts (https://www.hcharts.cn). All the tables were displayed based on DataTables (https://www.datatables.net). The BLAST function was established based on ViroBLAST (Deng *et al.* 2007). At the back-end, MySQL database system was used for storing the score data and the task details. AJAX was used for the communication between the front-end and the back-end.

## 3 Results

### 3.1 Data summary of C2CDB

**Figure 1.**
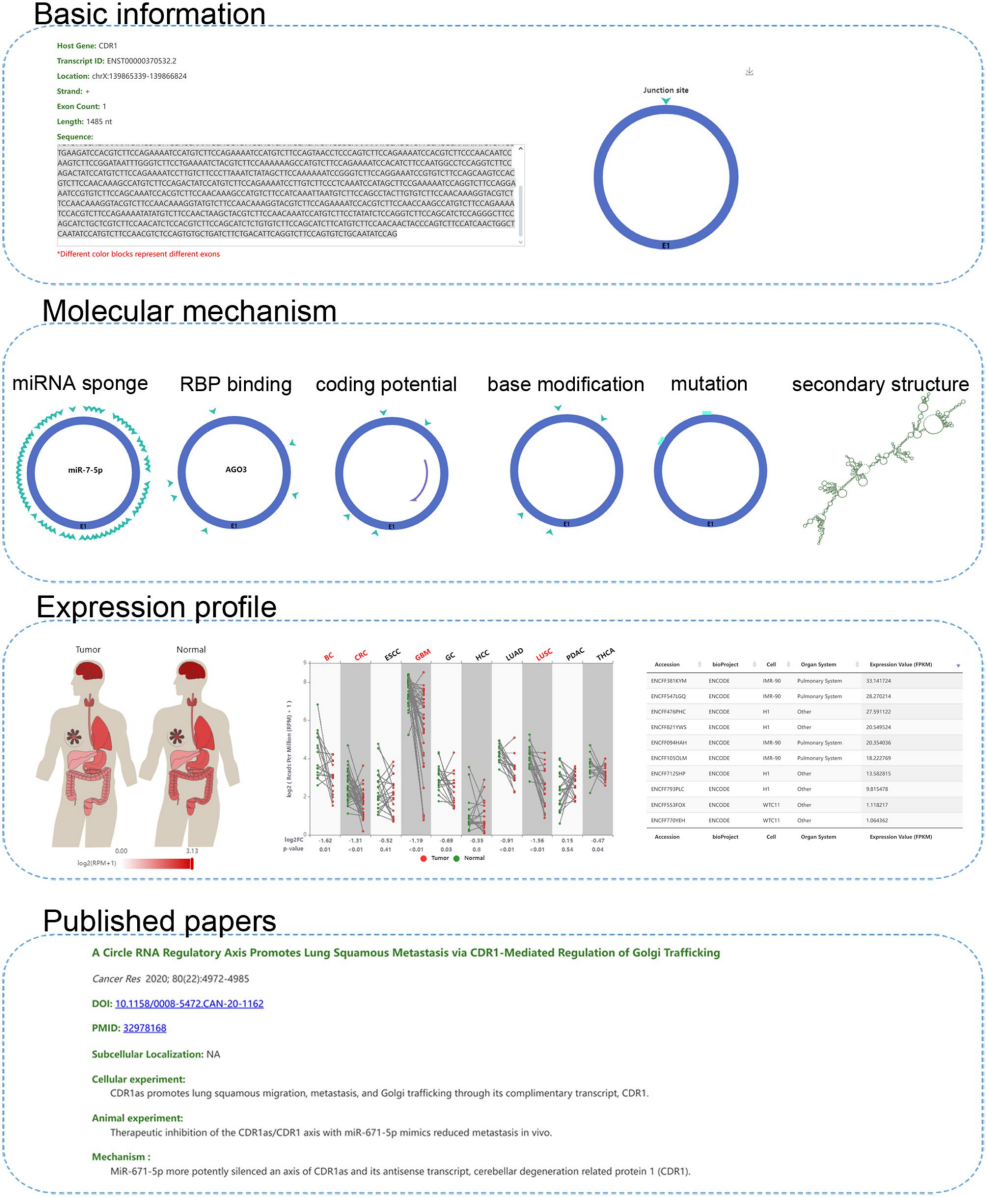
The data composition of C2CDB.

C2CDB quantified a total of 3 18 158 cancer-related circRNAs from 2510 cancer samples across 30 cancer types (Table 1), of which 96 597 were not included in any other database yet. Compared to existing circRNA databases, C2CDB includes a larger number of highly reliable circRNAs. Particularly, C2CDB has much more data and information than other four cancer-related circRNA databases (Table 2). Besides displaying basic information of circRNAs (such as host gene, genomic location, and sequence), C2CDB provides users elaborate information about the biological characteristics and molecular mechanism of cancer-related circRNAs, including miRNA sponge, RBP binding, coding potential, base modification (m^6^A, m^5^C, m^1^A, m^7^G and Psi), mutation (SNV), and secondary structure (Table 3). It covers almost all aspects of the information needed for current research on circRNA mechanism. Meanwhile, C2CDB displays the expression patterns of circRNAs and their host genes in different tumor and adjacent tissue from throughout available datasets, including data from West China Hospital and GEO database, as well as different tissues/cells and cancer cell-lines from ENCODE and GEO database. Importantly, C2CDB collected the experimental data of circRNAs from published articles, including biological function, mechanism, and molecular tools like RT-qPCR primers, Northern blot probes, and siRNA/shRNA sequences (Fig. 1).

**Table 1.** Summary of cancer samples of C2CDB database.

Cancer type	No. of datasets	No. of samples
Bladder cancer	8	56
Breast cancer	10	162
Cervical cancer	8	68
Clear cell renal cell carcinoma	5	36
Collecting duct carcinoma	1	12
Colorectal cancer	17	228
Distal cholangiocarcinoma	1	12
Esophageal squamous cell carcinoma	7	132
Gallbladder cancer	3	34
Gastric cancer	15	136
Glioblastoma	3	88
Hepatocellular carcinoma	19	492
Hypopharyngeal cancer	2	14
Infantile hemangioma	1	8
Intrahepatic cholangiocarcinoma	1	14
Laryngeal squamous cell carcinoma	3	130
Leukemia	6	215
Melanoma	1	6
Multiple myeloma	1	6
Nasopharyngeal carcinoma	1	8
Non-small cell lung cancer	10	182
Oligodendroglioma	1	82
Oral squamous cell carcinoma	4	120
Ovarian cancer	1	6
Pancreatic ductal adenocarcinoma	4	108
Pheochromocytoma and paraganglioma	1	14
Prostate cancer	4	44
Retinoblastoma	1	6
Thyroid carcinoma	9	120
Wilms tumor	1	9

**Table 2.** Comparison of C2CDB to other circRNA databases.

Database	No. of circRNAs from human	No. of Samples	No. of Species	Cancer specific	miRNA sponge	RBP binding	Coding potential	Modification	Mutation	Structure	Expression	Visualization
C2CDB	318 158	2510	1	Yes	Yes	Yes	Yes	Yes	Yes	Yes	Yes	Yes
circBase	140 682	75	6	No	No	No	No	No	No	No	No	No
circAtlas	768 986	2674	10	No	Yes	Yes	Yes	No	No	Yes	Yes	No
circBank	140 790	78	1	No	No	No	Yes	Yes	Yes	No	No	No
circRNADb	32 914	11	1	No	No	No	Yes	No	No	No	No	No
CIRCpedia2	79 084	185	6	No	No	No	No	No	No	No	Yes	No
DeepBase3	142 404	46 384	4	No	No	No	No	No	No	No	Yes	No
exoRBase2	148	905	1	No	No	No	No	No	No	No	Yes	No
circNet	289 303	2732	1	Yes	Yes	Yes	Yes	No	No	No	Yes	No
MiOncoCirc	227 056	2093	1	Yes	No	No	No	No	No	No	Yes	No
CSCD2	1 013 461	1113	1	Yes	Yes	Yes	Yes	No	No	No	No	Yes
CircRic	92 589	935	1	Yes	Yes	Yes	No	No	No	No	Yes	No
circMine	136 871	1107	1	No	Yes	No	No	No	No	No	Yes	No
circExp	Unknown	Unknown	1	No	No	No	No	No	No	No	Yes	No

**Table 3.** Statistics of circRNAs with biological traits in C2CDB.

	No. of circRNAs	Percentage
circRNA with miRNA binding sites	262 990	82.66
circRNA with RBP binding sites	287 394	90.33
circRNA with ORFs	22 698	7.13
circRNA with IRES	363	0.11
circRNA with modification site	175 679	55.22
circRNA with base mutation	276 513	86.91

### 3.2 Browse and search functions of C2CDB

#### 3.2.1 Search

In order to fulfill different users’ requirements, C2CDB provides users with three basic ways to query circRNAs in the module “Search”. First, users can search for circRNAs by any IDs from C2CDB or other seven databases (including circBase, circAtlas, circBank, circRNADb, DeepBase3, exoRBase2, and CIRCpedia2) or aliases from published articles. Second, users can search for miRNA to obtain circRNAs that binding to it. Third, C2CDB also allows users to query circRNAs by searching the interacting RBP.

#### 3.2.2 Genome Browser

The “Genome Browser” module is designed to provide users with a visual platform to explore circRNAs on the genome. Users can input specific genomic coordinates to search for circRNAs within a given region. Additionally, this module allows for a more intuitive understanding of circRNA’s exon composition, sequence direction, and other related features.

#### 3.2.3 BLAST

The BLAST tool provides users with the ability to search for circRNAs using sequence similarity. With this tool, users have the option to either upload a local FASTA file or directly input sequences into the designated box. After uploading the sequence, users can customize parameters such as mismatch scores, gap costs of matches, and the statistical significance expectation threshold in order to obtain the desired output. Additionally, users can apply filters to the results based on the BLAST score or similarity percentage of the sequences.

### 3.3 Obtain differentially expressed circRNAs in cancer

**Figure 2.**
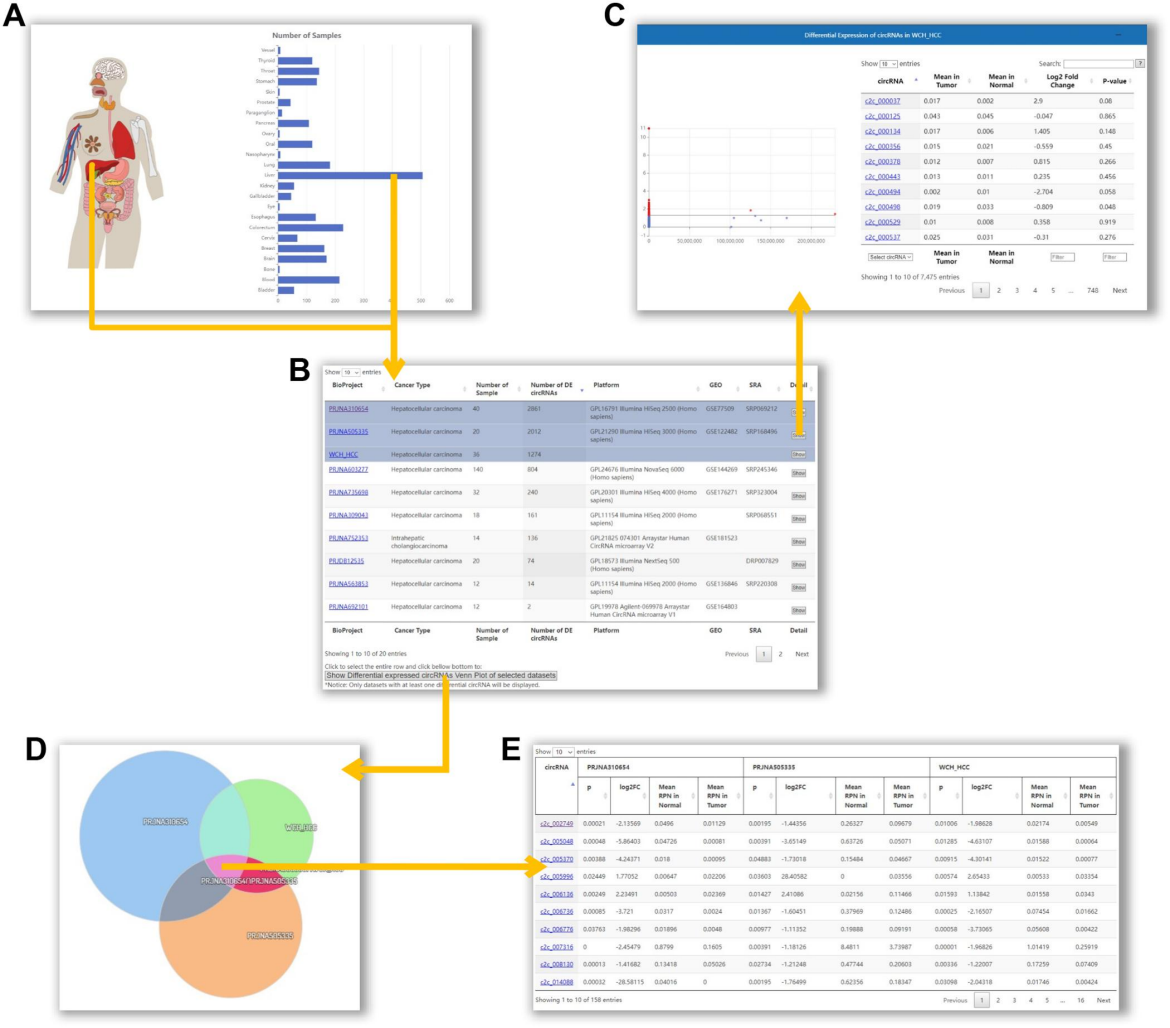
Utility of the “CircRNA in Cancer” module. (A) The page of “CircRNA in Cancer” module. (B) The table showing the list of datasets for the tissue selected in (A). (C) The list of differentially expressed circRNAs in the selected dataset. (D) Venn diagram illustrating the relationships between differentially expressed circRNAs from each selected dataset. (E) List of differentially expressed circRNAs commonly across multiple datasets.

To help users screen for differentially expressed circRNA candidates in cancer, C2CDB provides the “CircRNA in Cancer” module (Fig. 2). To begin, users can choose the organ they are interested in and access a list of tumor datasets for the selected organ. Upon clicking on the “show” button, users will see the list and volcano plot of differentially expressed circRNAs for that dataset. For screening differentially expressed circRNA candidates across multiple datasets, users can select one or more datasets and click on the “Show Differentially expressed circRNAs Venn Plot of selected datasets” button. This will display a Venn diagram illustrating the distribution relationships between differentially expressed circRNAs from each dataset. Users can then click on the overlapped area to view a list of differentially expressed circRNAs commonly across multiple datasets. Users can further filter the circRNAs based on information such as *P* value, fold-change, and mean value in cancer/normal displayed in the table. This allows users to identify their circRNAs of interest for further research.

### 3.4 Convert IDs of circRNAs across different databases

**Figure 3.**
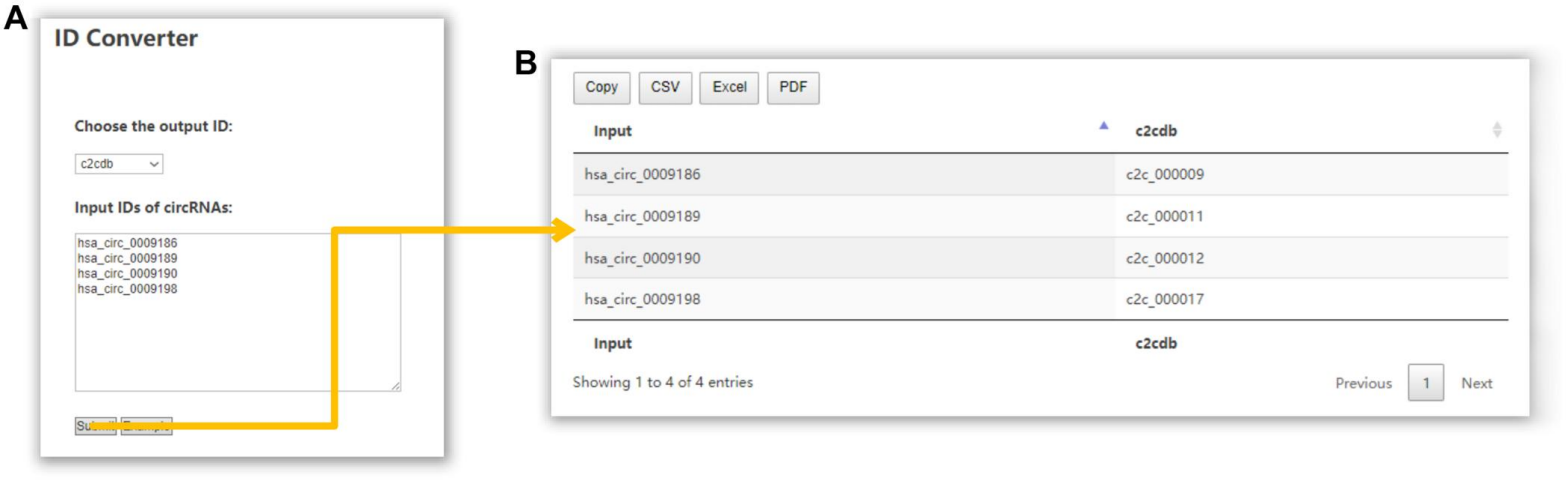
Utility of the “ID Converter” module. (A) The page of “ID Converter” tools. (B) The result page of “ID Converter” tools.

The nomenclature of a certain circRNA is not universal across databases and articles. For example, the well-known circRNA CDR1as is named as hsa_circ_0001946 in circBase, hsa_circCDR1_001 in circBank, hsa-circRNA8162 in DeepBase3, as well as circRNA7 and ciRS-7 in different articles, which may make the researchers confused. Given this, C2CDB provides users a new naming system of circRNAs that integrates the names of circRNAs from different databases and articles. Using the “ID Converter” tool, users can convert names of circRNAs across different databases (Fig. 3). In detail, users could input the list of circRNA IDs to be converted, choose the type of output ID, and click “Submit” to initiate the conversion. At the same time, in order to make the database more user-friendly, when querying circRNA using search tool and filtering the circRNA in any tables, circRNA names from different sources are allowed.

### 3.5 Integrated analysis to explore circRNA mechanisms

#### 3.5.1 circRNA–miRNA ceRNA interaction

**Figure 4.**
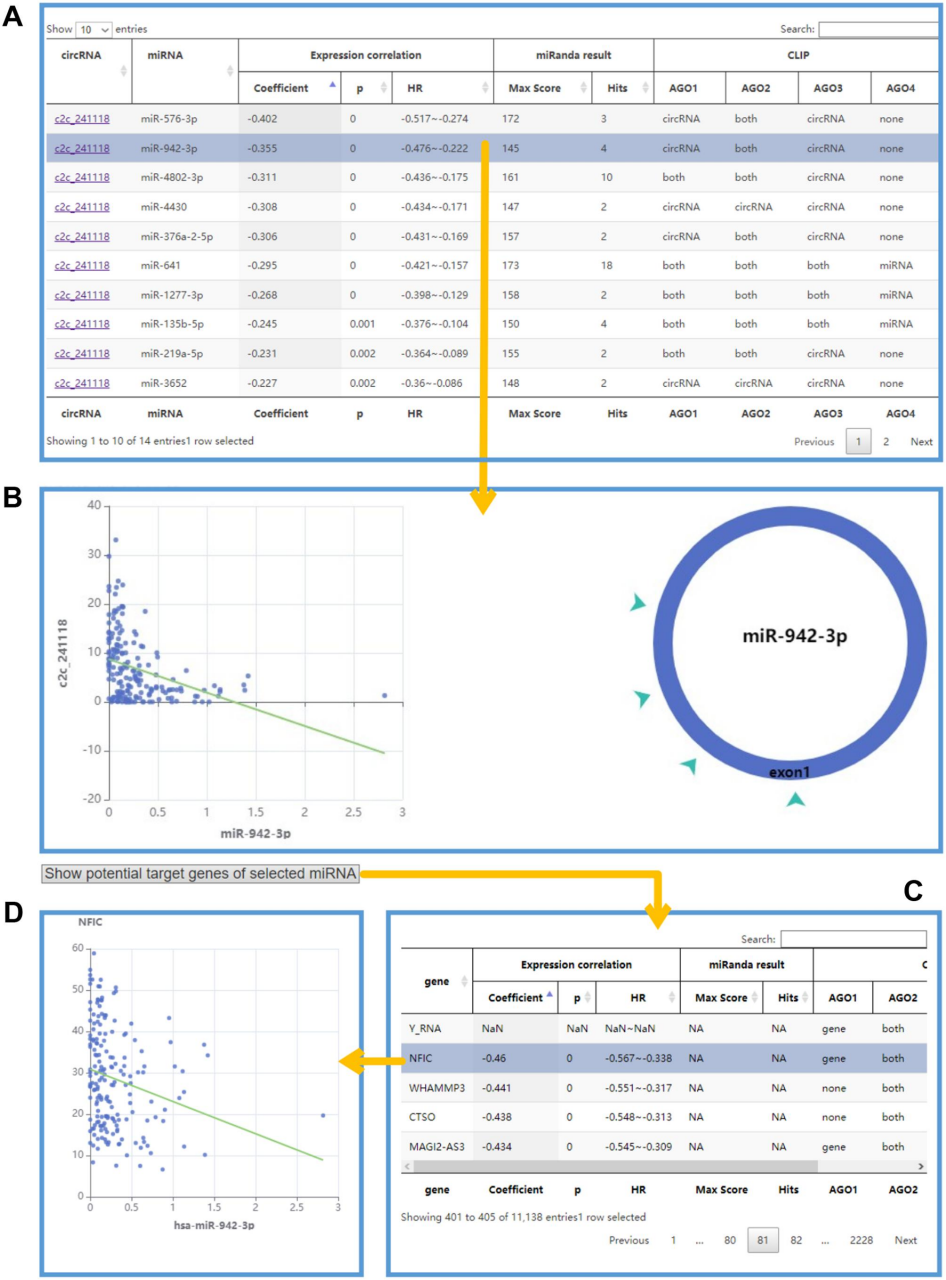
The result page of the “circRNA-miRNA ceRNA interaction” section. (A) The result table showing circRNA-miRNA pairs after analysing. (B) When clicking a row in the table, the information about the expression correlation and the diagram between circRNA and miRNA. (C) The part showing the potential target genes of miRNA selected in (A). (D) The expression correlation plot of miRNA and the selected target gene.

The “circRNA-miRNA ceRNA” section of C2CDB offers a range of analytical tools to explore circRNA–miRNA interactions (Fig. 4). Users can input the circRNA ID, select optional thresholds, and submit their query to obtain a list of circRNA-related miRNAs. Conversely, they can input a miRNA to acquire its target circRNAs. If users do not have a specific research target, they can select thresholds to retrieve potential miRNA–circRNA interacting pairs. The results page displays a table of circRNA–miRNA correlations that presents results from three aspects: expression correlation between circRNAs and miRNAs (including Pearson correlation coefficient, *P* value, and 95% confidence interval), miRanda prediction results (including max score and hits of circRNA-miRNA binding), and Ago-CLIP detection output (either/both of circRNA-miRNA pairs are detected from CLIP-seqs of Ago1, Ago2, Ago3, and Ago4 proteins). Clicking a row of the table will display the expression correlation plot of circRNA and miRNA, as well as a diagram of miRNA binding sites on circRNA. In addition, if users want to further investigate potential target genes of a selected miRNA, they can click the “Show potential target genes of selected miRNA” button. This will display a list of potential target genes of the miRNA.

#### 3.5.2 circRNA encoding

**Figure 5.**
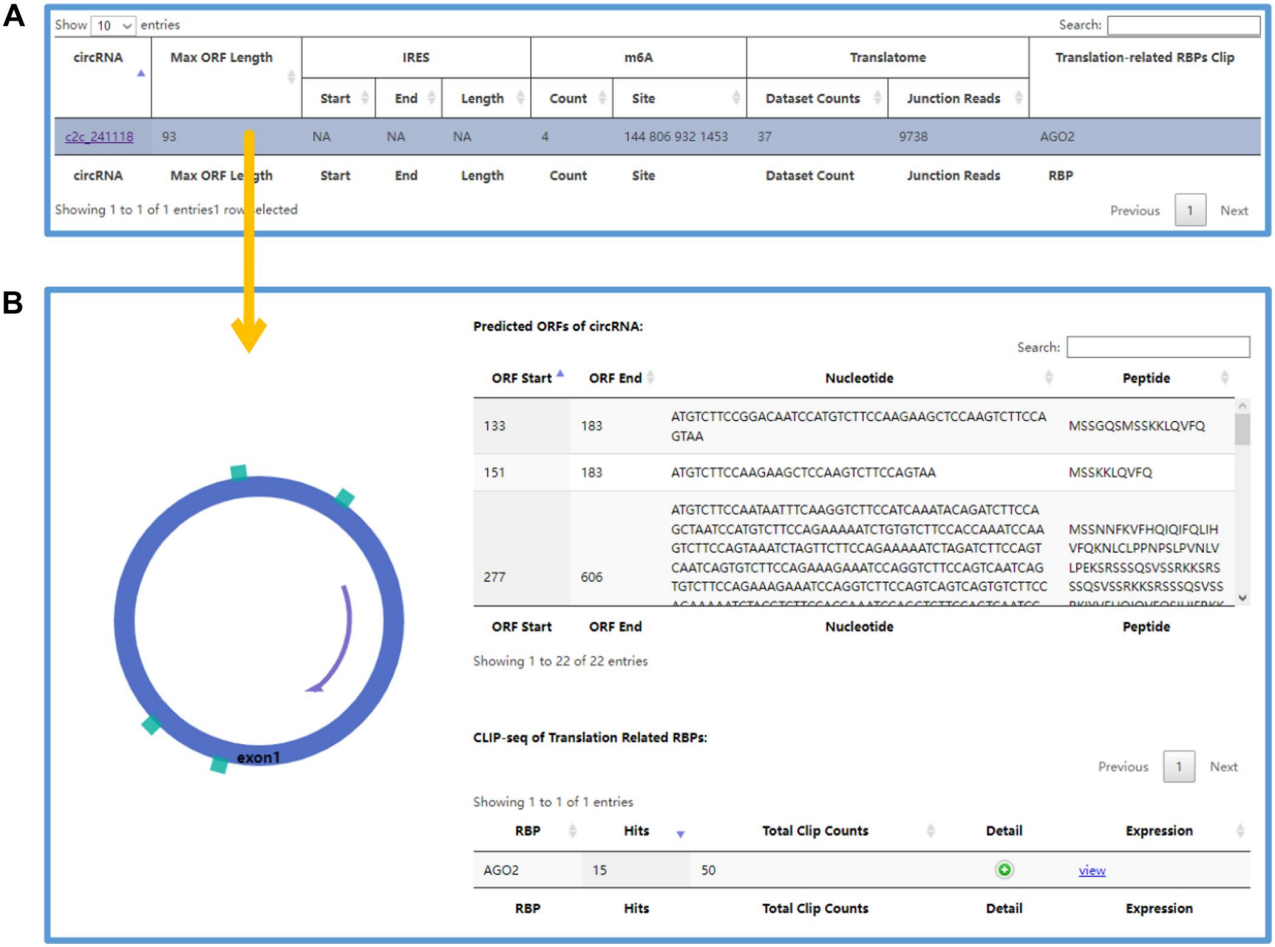
The result page of the “circRNA encoding” section. (A) The result table of circRNAs after analysing. (B) The detailed information about the coding potential of a certain circRNA.

The “circRNA encoding” section of C2CDB offers several features to help users investigate the potential for circRNAs to encode peptides (Fig. 5). First, users can input a circRNA ID to determine whether it has the ability to encode peptides. Second, if a user wants to identify which circRNAs are potentially regulated by a specific translation-related RBP, they can select this RBP and submit their query. Additionally, users can screen circRNAs based on their encoding-related characteristics, such as the presence of IRES element or m^6^A modification sites. After analysis, a table is generated that displays information on the ORF, IRES element, m^6^A modification site, detected reads in the translatome, and RBPs identified by CLIP-seq. Users can click on any row in the table to view detailed information and schematic diagrams related to the coding potential of the circRNA.

#### 3.5.3 circRNA biogenesis

**Figure 6.**
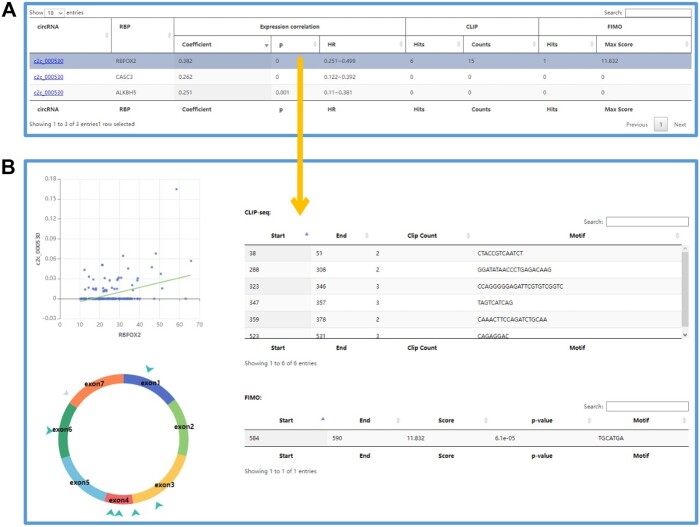
The result page of the “circRNA biogenesis” section. (A) The result table showing circRNAs and the RBP involving their biogenesis. (B) The detailed information about the expression correlation and the diagram between circRNA and the biogenesis related RBP.

The “circRNA biogenesis” section of C2CDB provides an array of analytical tools to facilitate circRNA research (Fig. 6). Users can perform analyses for diverse purposes, such as identifying RBPs that may regulate the biogenesis of their interested circRNAs. To do so, they can input the circRNA ID, apply various optional filters (such as correlation coefficient between the circRNA and RBP, supported by CLIP-seq and/or FIMO prediction), and submit their search. Alternatively, users have the option to explore which circRNAs may be under regulation by a particular RBP. This can be achieved by selecting the RBP of interest, configuring preferred filters, and then submitting a query for analysis. In case users only want to explore circRNA-RBP pairs meeting a specific threshold, they can adjust the filters and then browse the list of pairs. Upon completing the analysis, the results are presented in a table that displays expression relationships between circRNAs and RNA processing-related RBPs, as well as the circRNA-RBP binding detected from both RBP CLIP-seq and FIMO prediction. By clicking on a specific row, users can access more detailed information about the result. Additionally, the expression correlation plot between circRNA and RBP, as well as a diagram depicting circRNA-RBP binding, will be displayed for further exploration.

## 4 Discussion

In light of rapid advancements in circRNA research, it is crucial to stay updated with the latest progress and database developments. To meet this need, we have developed a cancer-specific circRNA database called C2CDB. C2CDB offers comprehensive information on expression profiles and biological mechanisms of cancer-related circRNAs. At the same time, C2CDB enables the interactive visualization to show the multidimensional information of biological mechanism of circRNAs. Besides analysing our own high-throughput RNA-seq data and other publicly available sequencing and microarray data, C2CDB also integrates published literatures to expand the experimental data about biological functions and mechanisms of circRNAs. Importantly, C2CDB established a comprehensive unified nomenclature system that matches the aliases and IDs of circRNAs from different databases and published articles. Notably, C2CDB integrates a vast collection of multi-omic sources, enabling comprehensive analysis functions that assist users in candidate selection, biogenesis studies, and investigations into the biological mechanisms of circRNAs. In summary, C2CDB is a valuable resource that not only provides information on the expression profiles, mechanisms, and functions of circRNAs in cancer, but also offers powerful tools for advanced circRNA analysis. Thus, we believe that C2CDB will greatly facilitate efficient utilization of public RNA-seq resources and serve as a useful web server for mining circRNAs in both tumor and normal samples. In future, more cancer-related circRNAs will be identified and their involvement in tumorigenesis will be elucidated. Especially, circRNAs exhibit promising potentials as clinical biomarkers and therapeutic targets. In the future, we would like to incorporate such experimentally validated circRNA information in the upgraded version of C2CDB, particularly focusing on clinical applications of circRNAs.

## Supplementary Material

vbae112_Supplementary_Data

## Data Availability

The data of this study are available at http://pengyonglab.com/c2cdb.
